# Safety and Effectiveness of Evolocumab During Acute and Sub-acute Phases of Acute Coronary Syndrome (ACS): A Systematic Review and Meta-analysis

**DOI:** 10.7759/cureus.35514

**Published:** 2023-02-27

**Authors:** Abdullah F Alzarroug, Haif K Al Gahtani, Saad Algahtani, Hatan K Alghamdi, Mohammad F Alhinti, Khalid A Almutairi, Sara Algahtani

**Affiliations:** 1 College of Medicine, Imam Mohammad Ibn Saud Islamic University, Riyadh, SAU; 2 Adult Cardiology, King Khalid University Hospital, King Saud University, Riyadh, SAU

**Keywords:** evolocumab, acute coronary syndrome, low-density lipoprotein cholesterol, adverse cardiovascular events, proprotein convertase subtilisin/kexin type 9

## Abstract

Background: Coronary artery disease (CAD), manifested mainly as acute coronary syndrome (ACS), continues to be a major cause of mortality globally and a significant contributing factor to the global disease burden. Elevation of low-density lipoprotein cholesterol levels attributed to proprotein convertase subtilisin/Kexin type-9 (PCSK9) during and following ACS puts patients at high risk of subsequent adverse events. Evolocumab is a PCSK9 inhibitor that is associated with a significant reduction in low-density lipoprotein cholesterol (LDL-C) levels through PCSK9 inhibition in comparison to traditional statin therapy.

Methods: We conducted a systematic review and meta-analysis of literature addressing the efficacy and safety of evolocumab compared to other lipid-lowering therapies or placebo. An extensive internet-based literature search using pre-determined key phrases supported by medical sub-headings and Boolean operators was performed in October 2022 to identify literature pertinent to the research topic. The search was primarily based on the National Library of Medicine (PubMed and Clinical Trials), MEDLINE, Cochrane, and the Science direct literature databases. Subsequently, the researchers devised PICOs-based screening criteria which had to be met by each identified study for inclusion in the review and meta-analysis. Two independent reviewers conducted data stratification and quality assessment of identified studies. Statistical analysis of the primary and secondary outcomes was conducted on the Cochrane REVMAN 5.4 statistical software for randomized trials.

Results: Two thousand five hundred and seventy-six potential studies were identified for inclusion in the systematic review. Data stratification, screening, and quality assessment of these studies based on the eligibility criteria led to the exclusion of two thousand five hundred and sixty-seven studies as they did not meet the standards set. Nine randomized controlled trials progressed to numerical analysis for validity and reliability. Eight studies were included in the meta-analysis. Meta-analytical results showed a significant decrease in LDL-C changes from initiation of evolocumab therapy to 8 weeks following ACS compared to placebo. Similar results were derived in the sub-acute phase of ACS [SMD -1.95 (95% CI -2.29, -1.62)]. The meta-analysis revealed no statistically significant relationship between the risk of adverse effects, serious adverse effects, and major adverse cardiovascular events (MACE) from treatment using evolocumab in comparison to placebo [(relative risk, RR 1.04 (95% CI 0.99, 1.08) (Z = 1.53; p=0.12)].

Conclusion: Early evolocumab therapy initiation was associated with a significant decrease in LDL-C levels and was not associated with an increased risk of adverse effects in comparison to placebo.

## Introduction and background

Coronary artery disease (CAD), defined by the World Health Organization, refers to a medical condition characterized by inadequate blood and oxygen supply to the cardiac myocardium. It has been articulated extensively as one of the leading causes of death globally. Studies attribute CAD to about two percent of the global disease burden [1]. Reducing low-density lipoprotein cholesterol (LDL-C) levels is a desired therapeutic target in the prevention or the risk reduction of adverse cardiovascular events [2-5]. Over the years, this task has been amicably served by moderate- or high-intensity statins [6]. However, based on recent findings associating statins with lipid reduction effects in less stable CAD patients has warranted further research on the efficacy of early initiation of statin therapy following ACS onset [7].

Proprotein convertase subtilisin/Kexin type-9 (PCSK9) has been articulated as the primary low-density lipoprotein metabolism regulator. Recent data showed that decreased mutation vigor of PCSK9 genes has been significantly associated with reduced LDL-C levels and the incidence of adverse coronary events. Thus, PSCK9 genes remain a significant therapeutic target [8]. Alirocumab and evolocumab are monoclonal-antibody-based PSCK9 inhibitors associated with long-term efficacy in reducing significant adverse cardiovascular events in patients with documented atherosclerosis-based cardiovascular disorders. Alirocumab and evolocumab inhibitors were approved following evaluations in the ODYSSEY (phase two) and PROFICIO (phase three) randomized clinical trials, respectively [9]. Recent studies have highlighted a significant efficacy in LDL-C reduction following evolocumab administration in ACS patients with the FOURIER clinical trial demonstrating substantial decreases in mortality ensuing from cardiovascular events, stroke, myocardial infarction (MI), and hospitalization due to adverse cardiac events [10-11].

Klassen et al. observed limited data on the efficacy of early initiation of evolocumab inhibitors before and after hospital discharge in ACS patients [12]. However, Ray et al. reported a significant efficacy of early statin therapy following ACS events as collaborated by trials by the PROVE-IT-TIMI 22 (Pravastatin or Atorvastatin Evaluation and Infection Therapy-Thrombosis in Myocardial Infarction-22) and the MIRACL (Myocardial Ischemia Reduction with Aggressive Cholesterol Lowering) [13-14]. These observations significantly influenced our endeavor to analyze the impact of early initiation of evolocumab in ACS events. Therefore, this review aims to statistically analyze the effectiveness of evolocumab treatment in addition to or in the absence of statin therapy against a placebo based on two distinct timeframes (acute and sub-acute phases) to determine the effectiveness of evolocumab administration at early stages of ACS.

Notably, this systematic review and meta-analysis observe the significant contributions made by a similar systematic review and meta-analysis conducted by Guedeney et al. (2022), on the roles of evolocumab and alirocumab PCSK9 inhibitors in the treatment of ACS [15]. Therefore, the review sought to provide a comprehensively detailed analysis of evolocumab as the sole PCSK9 inhibitor compared to outcomes presented by Guedeney et al. (2022) between two phases in the early treatment phases acute (one to eight weeks) and sub-acute (eight to 16 weeks).

## Review

Methods

Research Design

This systematic review and meta-analysis strictly adhered to the PRISMA 2021 guidelines constructed by Moher et al. [16]. Initially, an updated protocol was drafted and registered in the PROSPERO database and submitted to the National Institute for Health Research at the University of York Center for Reviews and Dissemination. To counter any possible conflicts of interest from other prospective authors (ID: CRD42022379669).

Research Objectives

The primary objective for performing this systematic review and meta-analysis was to derive and present clear and concise statistical outcomes on the efficacy of evolocumab in two treatment phases, the acute and sub-acute phases following ACS events in relation to reduction in LDL-C levels. Secondly, we sought to compare the adverse effects of evolocumab in comparison to placebo.

Literature Search Strategy

An extensive literature search for data relevant to the research question was digitally conducted in medical databases in October 2022. The reviews employed a pre-determined search string generated from relevant keywords with a combination of medical subject headings (MeSH terms). The search string used Boolean operators, relevant truncations, and filled tags for maximum optimization of the search process.

The National Library of Medicine NIH library, hosting numerous medically relevant journal databases such as the PubMed and Clinical Trials databases, was utilized as the principal literature source. Additionally, the Cochrane Library for systematic reviews and meta-analysis, the Web of Science (WOS), and the MEDINE databases were extensively scoured to derive relevant literature to the research question. This extensive literature search was conducted to derive approved, peer-reviewed, and published papers for inclusion in the systematic review and meta-analysis. The systematic review and meta-analysis also wish to disclose additional data sources from scouring references of the retrieved relevant articles in search of additional literature for inclusion in the meta-analysis. Moreover, a thorough search of peer-reviewed but unpublished gray literature through a manual search of pertinent green papers was submitted to universities, company websites, and research centers.

Screening

To ensure only the most relevant articles and studies were included in the meta-analysis, the authors developed an eligibility selection criterion that each included report had to satisfy to be included in the meta-analysis, as highlighted by Stillwell et al. (2010) [17]. Consequently, the systematic review and meta-analysis included only studies meeting the specifications outlined below.

**Population (P):** Studies reported patients above 18 years were recently hospitalized for acute and sub-acute phases of ACS. The diagnosis for ACS was exclusively limited to patients exhibiting NSTEMI and STEMI myocardial infarction within 24 h of onset and unstable angina within 72 h of onset. Moreover, studies reported patients with elevated LDL-C levels (≥ 70 mg/dL) and/or treated with moderate-intensity statins (10 mg rosuvastatin or atorvastatin 20 mg) as per recommendations of the Expert Consensus on Clinical Pathway of Blood Lipid Management in Patients with acute coronary syndrome (ACS).

**Intervention (I):** Studies reported patients administered evolocumab within 72 h of ACS diagnosis. Evolocumab, commonly referred to by its tradename Repatha, is administered subcutaneously using a single prefilled auto-injector with a dosage of 140 mg.

**Comparison (C):** Studies reported changes in LDL-C levels on the administration of evolocumab from baseline after one and two weeks following acute and sub-acute phases of ACS compared to placebo or other lipid-level lowering drugs.

**Outcome (O):** Only studies reported changes in LDL-C levels from evolocumab administration as the primary outcome. Moreover, studies reported incidences of CAD mortality, myocardial infarctions, unstable angina, stroke, and unprecedented coronary revascularizations that will be included in the study.

**Others (O):** To ensure a precise representation of statistical analysis, the authors deemed it necessary to include studies dated from 2015 to 2022. Thus, chronological filters were also incorporated into the eligibility criterion. Additionally, language filters were included in the eligibility criterion to derive studies and articles in English.

Study Selection and Data Collection

Following the initial data search on the databases mentioned above, this systematic review and meta-analysis employed two independent reviewers to conduct title, abstract, and full-text analysis on the identified papers. All authors critically appraised the full-text analysis of records deemed fit for inclusion by one or all the reviewers. The principal researcher forwarded the resulting discrepancies, and disagreements were amicably resolved through cordial discussion. Moreover, consultations were made with statisticians and professionals in the research field to gain additional insights into the meta-analysis. Studies derived from the initial database search were recorded into an MS excel spreadsheet. A separate spreadsheet was utilized for data stratification when the identified records were screened using the eligibility criteria. A narrative table detailing study characteristics of individual studies was developed and cordially filled by the authors.

DATA Analysis

The Cochrane Tool for systematic reviews and meta-analysis of randomized trials, REVMAN 5.40, was utilized as the main data analysis tool. Continuous outcomes reported in individual studies as means and standard deviations were analyzed using a random effects model into standard mean differences (SMDs) using a 95% CI. Dichotomous outcomes were analyzed using a fixed effects model into odd ratio (OR) using a 95% CI. Heterogeneity between the studies was calculated using the Tau-square and the I2. The p values lower than 0.05 (p<0.05) were considered statistically significant.

Quality Appraisal

As per the PRISMA 2021 guidelines, resulting studies deemed fit for inclusion in the systematic review and meta-analysis were extensively assessed by independent reviewers for the resulting risk of bias. This systematic review and meta-analysis utilized the Cochrane quality assessment tool (ROB-2), recommended by Higgins et al. [18]. Only studies exhibiting a low risk of bias were included in the review and meta-analysis.

Results

Literature Search

**Figure 1:**
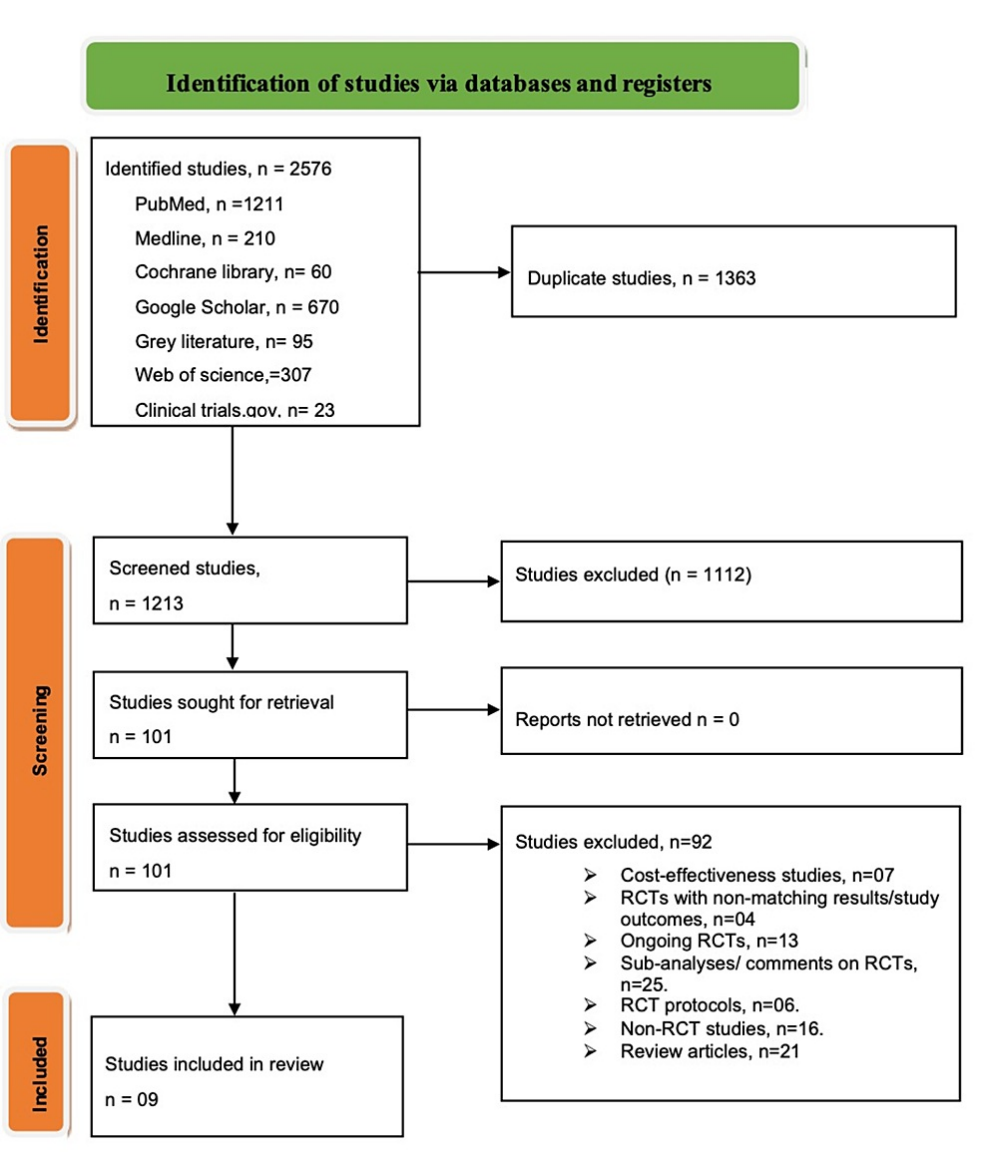
Pathway of the identification of studies via databases and registers.

The initial database search derived 2576 studies. Subsequent stratification from abstract and title scrutiny led to the expulsion of 1363 duplicate records. Some 1112 studies were excluded following in-depth abstract and title scouring; 16 studies did not have abstract, while the exclusion of 1096 studies was based on incompatibility with this meta-analysis's objectives and poster presentations and non-English. Finally, 101 studies proceeded for eligibility screening. Consequently, 92 studies were deemed unfit for inclusion in the review based on; seven studies were oriented on assessing cost-effectiveness, four randomized controlled trials (RCTs) reported non-matching patients' outcomes, 13 studies were ongoing trials, 25 studies were sub-analyses/comments on RCTs, six studies were only RCT protocols, 16 studies were non-randomized clinical trials while 21 studies were review articles. Nine studies proceeded for quantitative test accuracy for reliability and validity (Figure 1).

Study characteristics

The characteristics of the studies included in the meta-analysis are shown in Table 1.

**Table 1:** Characteristics of the included studies. *According to the China Cholesterol Education Program Expert Consensus, the therapeutic LDL-C concentration target for patients at exceptionally high risk should be lower than 55 mg/dL or achieve at least a 50% reduction from baseline. LDL-C, low-density lipoprotein cholesterol

STUDY ID	Design	Trial ID	Population	Treatment period (weeks, wks)	Primary outcomes assessed	Outcomes
Konstantinos et al. [19]	Randomized controlled study	NCT03287609	308	Eight wks	Changes in LDL-C from baseline to 8 weeks	Following eight weeks of evolocumab treatment, the mean LDL-C levels decreased significantly (from 3.61 mmol/L to 0.79 mmol/L) compared to 3.42 to 2.06 mmol/L in the placebo group
Li et al. [20]	Randomized controlled study	Approved by the Medical Ethics Committee of Tianjin Chest Hospital	99	Eight wks	Changes in LDL-C from baseline to 8 weeks	After an 8-week treatment period, 96.3% of patients in the evolocumab achieved the therapeutic target, while only 13.3% in the control group achieved the therapeutic target.*
Sabatine et al., [21]	Randomized controlled study	NCT0185418 (phase 2). NCT101439880 (phase 3)	4465	12 wks	Changes in LDL-C from baseline to 12 weeks	A 61% reduction in LDL-C levels was realized when ACS patients were treated with evolocumab. Additionally, the incidence of cardiovascular events after one year was significantly reduced in the evolocumab group (2.18% to 0.47% compared to 2.18 % to 0.95% in the standard therapy.
Nakamura et al. [22]	Randomized controlled study	Approved by the Iwate Prefectural Central Hospital, Japan	36	Three wks	Changes in LDL-C from baseline to 20 days	Plasma, LDL-C in placebo did not increase significantly from baseline, whereas in the evolocumab, a continuous decrease was observed after 20 days.
Hao et al. [23]	Randomized controlled study	/	136	Four wks (12 wks follow-up)	Changes in LDL-C from baseline to 4 weeks	Early intervention with evolocumab in patients at high risk of ACS with high LDL-C levels resulted in rapid cholesterol and lipid-lowering and improved cardiovascular prognosis.
Nicholls et al. [24]	Randomized controlled study	NCT01813422	968	78 wks	Primary atheroma volume	The study found that adding PCSK9 inhibitor evolocumab to statin therapy reduced LDL-C levels and subsequent atheroma regression.
Vavuranakis et al. [25]	Randomized controlled study	NCT03515304 & NCTO4O82442	74	Four wks	Changes in LDL-C from baseline to 8 weeks	LDL-C levels increase during ACS events; however, early treatment with evolocumab was shown to decrease these levels after 24 h of hospital admission.
Leucker et al. [26]	Randomized controlled study	NCTO3515304	272	Four wks	Changes in LDL-C from baseline to 8 weeks	Evolocumab administration in the intervention group resulted in a significant difference in LDL-C levels after three days compared to the placebo group. Similar results were observed after a 30-day follow-up period.

Meta-analysis Outcomes

 A total of eight studies were included in the meta-analysis of observed statistical outcomes expressed in the individual studies. The primary outcomes assessed by the meta-analysis were changes in LDL-C levels from baseline to eight weeks (acute) and changes from baseline to sub-acute (more than eight weeks) following evolocumab and placebo initiation in the studies. At the same time, the secondary outcomes included the prevalence of severe and adverse events following therapy initiation. Statistical analysis was conducted using Cochrane REVMAN 5.4 software using a random effects model.

**Figure 2:**
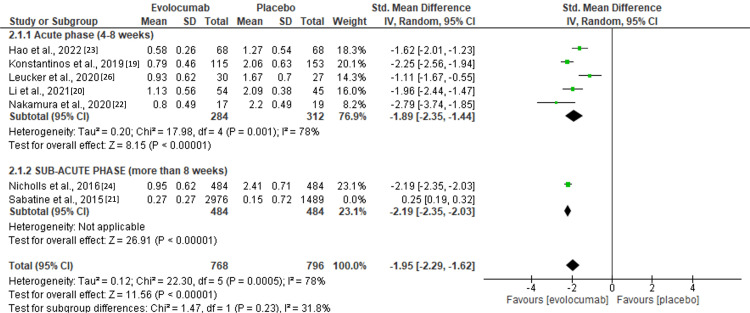
Forest plot showing changes in LDL-C levels following evolocumab initiation. Source: [19-24, 26] LDL-C, low-density lipoprotein cholesterol

**1.** **Primary outcome (LDL-C changes from baseline):** statistical outcomes were represented in terms of mean ± standard deviations (SDs) mmol/L. To convert mg/dL to mmol/L, multiply by 0.02586 (Figure 2).

**Figure 3:**
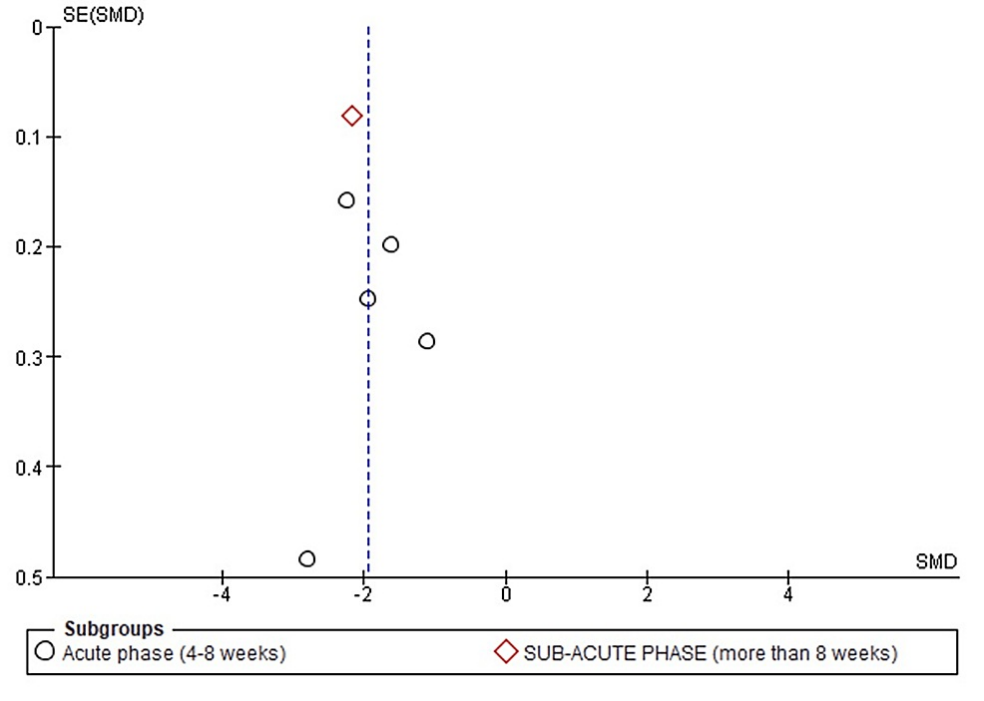
Funnel plot showing changes in LDL-C levels following evolocumab initiation. LDL-C, low-density lipoprotein cholesterol

The result shows a significant, p < 0.05, decrease in LDL-C values following evolocumab initiation [standardized mean difference, SMD -1.95 (95% CI -2.29, -1.62)] compared to placebo. However, high heterogeneity was observed in the subsequent meta-analysis, I2 =78%. This observation was accredited to differences in periods of study where measured data were collected (Figure 3).

**Figure 4:**
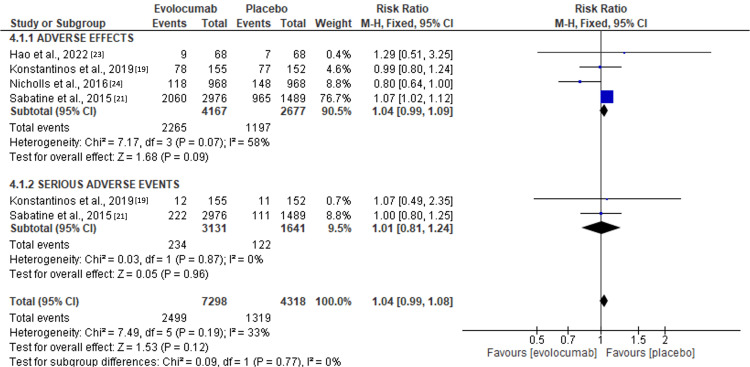
Forest plot showing the incidence of adverse outcomes following evolocumab initiation. Source: [19, 21, 23-24]

**Figure 5:**
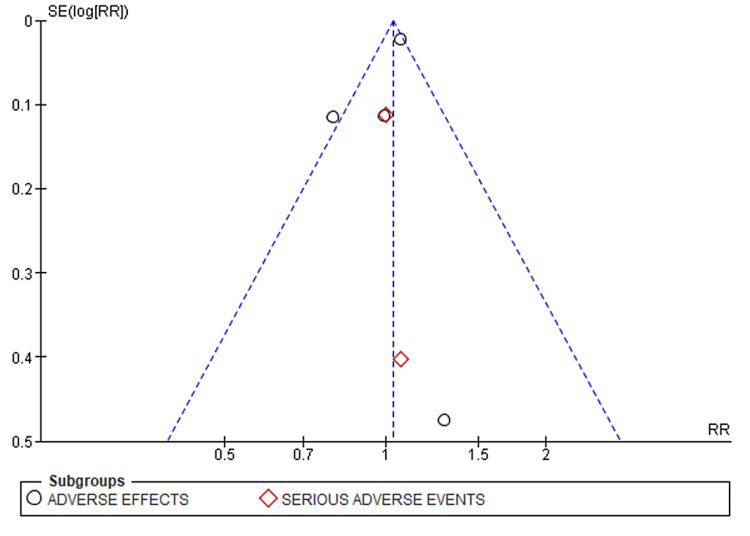
Funnel plot showing the distribution of bias derived from individual studies documenting the incidence of adverse outcomes following evolocumab initiation.

**2. Secondary outcome (risk of adverse events):** all studies reported a significant prevalence of adverse effects in the treatment and control groups. However, compared to the placebo, the risk of adverse and serious effects from treatment was relatively higher in the evolocumab group in the individual studies. A meta-analysis based on the dichotomous outcomes realized revealed a [RR 1.04 (95% CI 0.99, 1.08)] though statistically insignificant (Z=1.53; p=0.12). Thus, no association between evolocumab and adverse effects, whether severe or mild, was derived from the meta-analysis. A relatively moderate heterogeneity (I2 =33%, Chi 2=7.49) was observed between the studies (Figures 4-5).

Risk of bias

**Figure 6:**
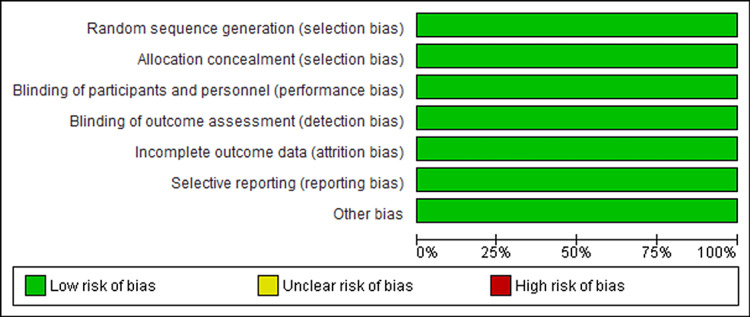
Risk of bias graph.

**Figure 7:**
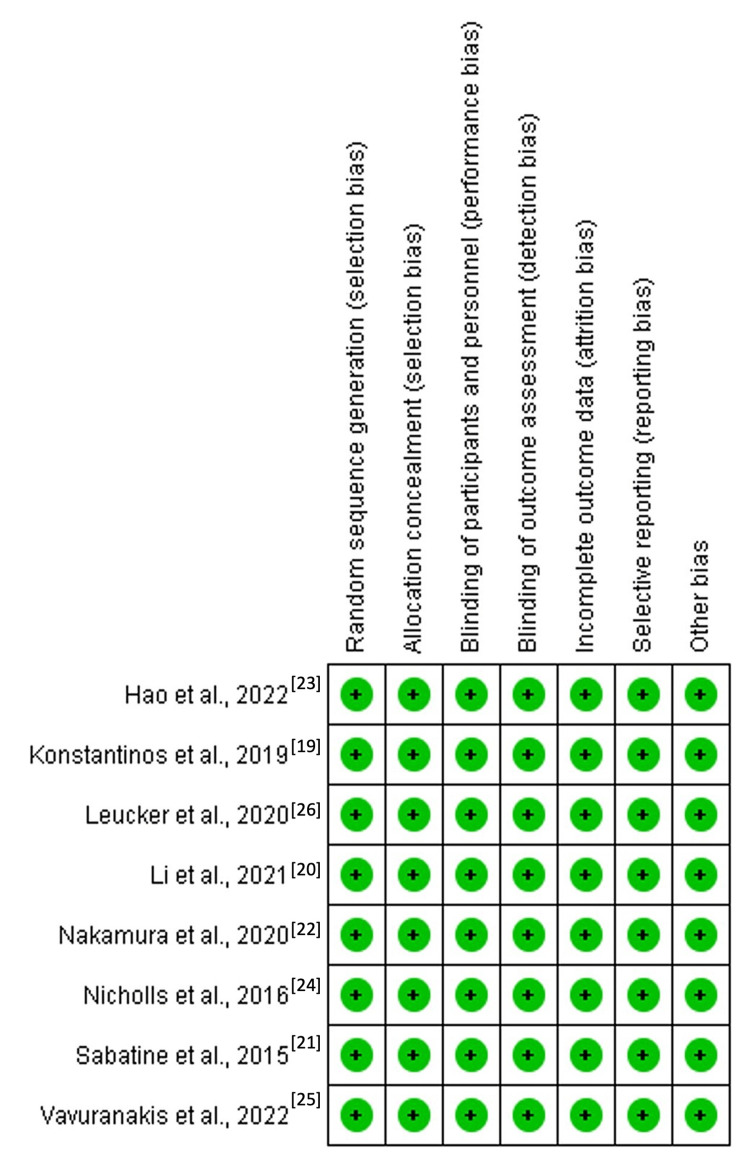
Risk of bias summary. Source: [19-26].

Following a quality appraisal of individual studies using the Cochrane Risk of bias tool, this systematic review and meta-analysis found that each study posed a low risk of bias. This observation was accredited to the double-blinding of patients and clinicians. Moreover, all studies reported a randomized selection of patients. The summary of the risk of bias and the graph representing the risk of bias for each study are outlined in Figures 6-7 below.

Discussion

Nine randomized and relevantly approved randomized controlled trials detailing evidence realized from the application of evolocumab in patients diagnosed with ACS in either the sub-acute phase or the acute phase were included in this systematic review and meta-analysis. As per the authors’ judgments, the sub-acute phase was defined as the time of application of evolocumab to eight weeks, while the acute phase was evolocumab application after eight weeks in the ACS population. A significant reduction in LDL-C levels was observed in all phases (acute or sub-acute) following the evolocumab intervention, p < 0.5 [SMD (-1.95 (95% CI -2.29, -1.62)]. These findings complement a recent meta-analysis conducted by Guedeney et al. [15]. Seven studies [19-20, 22-23, 25-26] assessing LDL-C reduction outcomes from baseline to eight weeks, the sub-acute phase, revealed a significantly positive association between LDL-C reduction and the application of evolocumab [p < 0.5, (SMD, -1.89 (95% CI -2.35, -1.44)]. Two studies, Sabatine et al. and Nicholls et al. also revealed similar statistical LDL-C reduction levels when compared with placebo, p < 0.5, [SMD (-2.19, 95% CI -2.35, 2.03)] showing a higher LDL-C reduction rate in the acute phase when compared to control [21, 24]. However, based on the limited number of studies, two, the authors deemed this result inconclusive and recommended more studies assessing the efficacy of evolocumab in the long term. Overall, the review finds that the early administration of evolocumab, either in the acute or sub-acute phases, was significantly associated with a significant reduction in LDL-C values when compared to placebo, p < 0.5 [SMD (-1.95 (95% CI -2.29, -1.62)].

Unfortunately, approximately 20% of ACS survivors have been associated with subsequent ischemic cardiovascular events within 24 months and five years. The reported mortality rates range from 19% to 22% [27-28]. In addition, the recurrence of ACS has been significantly associated with increased mortality [29]. Therefore, the review sought to analyze the risks of adverse effects and major adverse cardiovascular events (MACE) on surviving ACS patients treated with evolocumab at early stages. Similar to the findings observed by Guedeney et al. [15], significant ischemic endpoints were observed following the initiation of evolocumab in ACS patients. Adverse events and MACE outcomes were well represented in the evolocumab and placebo groups. A meta-analysis of continuous outcomes revealed that compared to placebo, the risk of adverse events was statistically insignificant in the evolocumab treatment group {RR [1.04, (95% CI 0.99, 1.09)]} p=0.09. Similarly, compared to the placebo, evolocumab showed a statistically insignificant association regarding MACE rates [RR 1.01 (95% CI 0.81, 1.24)] p=0.96. The heterogeneities in the two outcomes were insignificant, p=0.07 and p=0.96, respectively. Thus, the results obtained by the review show the significant role played by PCSK9 inhibitors in the reduction of MACE in the treatment groups, similar to findings derived by Guedeney et al. [15]. These findings on the insignificant association of evolocumab with adverse and MACE outcomes align with conclusions derived from the GLAGOV trial, where ACS patients treated with statins had significant reductions in the rates of plaque regressions and overall atheroma volumes [24]. Some of the most frequently encountered mild adverse events in the evolocumab group were arthralgia, headaches, muscle and joint aches, fatigue, pain at the injection sites, influenza, and coughs [21]. Only two studies reported on MACE, Sabatine et al. and Konstantinos et al. with higher levels of major coronary events (coronary revascularization) observed in the two comparison groups [19, 21]. Major adverse cardiovascular Events observed in the studies included death; major coronary events, including myocardial infarction, unstable enigma, and coronary revascularization; and cerebrovascular events, which were largely composed of stroke and transient heart attacks were observed in both evolocumab and the control groups. Cardiovascular deaths were relatively low, with only 15 documented deaths from the abovementioned studies. Compared to the placebo, the rates of MACE in the evolocumab group were relatively lower than in the placebo group [24].

Dubuc et al. hypothesized that a combination of PSCK9 inhibitors and statin medications could improve therapeutic effects among ACS patients [30]. In addition, per guidelines stipulated for the management of post-ACS events, there is a paramount need to reduce low-density lipoprotein cholesterol levels [31]. Thus, clinicians and medical researchers developed monoclonal PSCK9 inhibitors. Monoclonal inhibitors are antibodies with high-mass proteins subcutaneously or intramuscularly injected, which have been documented to be effective in restricting the availability of PSCK9 binding sites preventing low-density lipoprotein receptors degradation resulting in LDL capture and elimination. Li et al. and Abtan et al. document that in the presence of ACS events, the first year is the most vulnerable for surviving patients, with about one in five patients experiencing a new event during the first year following the ACS event [20, 32]. This vulnerability is relatively high among older patients.

This systematic review and meta-analysis did not encounter significant limitations in the development period. However, several limitations were disclosed in individual studies deemed to influence prospective findings. Firstly, some studies highlighted low study populations -- Li et al., Nakamura et al., and Vavuranakis et al. [20, 22, 25]. Secondly, several limitations in the length of the study and follow-up period were reported in almost all studies except the Nicholls et al. study [24]. Thirdly, the review and meta-analysis wish to disclose challenges in data stratification since some studies reported outcomes of two phases of clinical trials [10, 25].

## Conclusions

Monoclonal PCSK9 inhibiting antibodies such as evolocumab or Repatha in combination with high-intensity statins or solely have been associated with a significant reduction in LDL-C levels from baseline compared to outcomes recorded from placebo analysis. The most significant changes in the LDL-C levels were observed in the acute phase following ACS. Both therapy and placebo groups reported a significant prevalence of adverse and serious adverse cardiovascular events after the occurrence of ACS. However, the prevalence of these cardiovascular events was higher in the placebo groups than in the evolocumab intervention groups and no association between evolocumab and adverse effects, whether severe or mild, was derived from the meta-analysis.
